# Supplementary material for: Levels of Evidence Supporting United States Guidelines in Pancreatic Adenocarcinoma Treatment
Source: Cancers (Basel). 2022 Aug 22;14(16):4062. doi: 10.3390/cancers14164062 (PMC9406577; doi:10.3390/cancers14164062)
Supplement: Supplementary file 1 [file cancers-14-04062-s001.zip › cancers-1857265-supplementary.pdf]

cancers MDPI

Supplementary Materials

Table S1. Data extraction form for therapeutic recommendations

	Recommendation
Location in the document (where?)	Text ☐<br>Summary table ☐<br>Algorithm ☐
Type of recommendation	Therapeutic ☐<br>Incidence and epidemiology ☐<br>Diagnostic and Staging ☐<br>Follow-up ☐
Disease stage	Potentially curable disease ☐<br>Advanced disease ☐<br>All stages ☐
Type of treatment	Surgery ☐<br>Chemotherapy ☐<br>Chemoradiotherapy ☐<br>Chemotherapy or chemoradiotherapy ☐<br>Combination of chemotherapy and chemoradiotherapy ☐<br>Palliative care (endoscopy, pain medication etc…) ☐<br>Other (clinical trials, experimental treatments, etc…) ☐
Classification of evidence	

A/Definitions for Quality of Evidence grades [1–8]

Table S2. Classification of evidence used by the American Society of Clinical Oncology (ASCO guidelines) based on the GRADE methodology (extracted from the ASCO guidelines methodology manual).

**GRADE**	**DEFINITION**
HIGH	We are very confident that the true effect lies close to that of the estimate of the effect.
MODERATE	We are moderately confident in the effect estimate: The true effect is likely to be close to the estimate of the effect, but there is a possibility that it is substantially different
LOW	Our confidence in the effect estimate is limited: The true effect may be substantially different from the estimate of the effect.
VERY LOW	We have very little confidence in the effect estimate: The true effect is likely to be substantially different from the estimate of effect

GRADE's approach to rating the quality of evidence begins with the study design (randomized trials versus observational studies) and then addresses five reasons to possibly rate down the quality of evidence and three to possibly rate it up [1]. The five reasons to possibly rate down the evidence is based on the evaluation of risk of bias [2], inconsistency of results [3], indirectness of evidence [4], imprecision [5] and publication

bias [6]. Factors that can increase the level of evidence include the magnitude of the effect, the dose response gradient and the effect of plausible residual confounding [7]. Review the quality of evidence for each pre-specified critical outcome. If the quality rating is the same for each outcome, the same is true for the overall quality of evidence [8]. If the quality rating differs, the lowest quality of evidence for any critical outcome determines the overall quality.

B/Strengths of recommendation [9]

The Expert Panel provides a rating of the strength of each recommendation. This assessment reflects the extent to which a guideline panel is confident that desirable effects of an intervention outweigh undesirable effects, or vice versa, across the range of patients for whom the recommendation is intended. Recommendations may fall into two categories; strong and weak. Factors determining the strength of a recommendation include balance between benefits and harms, certainty of evidence, confidence in values & preferences, and resource use [10]. Recommendations may be made for or against the use of an intervention ("strong for", "strong against", "weak for", "weak against").

Table S3: Classification of evidence used by the National Comprehensive Cancer Network guidelines (www.nccn.org).

**Category 1**	Based upon high-level evidence, there is uniform NCCN consensus that the intervention is appropriate.
**Category 2A**	Based upon lower-level evidence, there is uniform NCCN consensus that the intervention is appropriate.
**Category 2B**	Based upon lower-level evidence, there is NCCN consensus that the intervention is appropriate.
**Category 3**	Based upon any level of evidence, there is major NCCN disagreement that the intervention is appropriate.

As mentioned on www.nccn.org (development and update of guidelines), the level of evidence depends upon the following factors: quality of data (e.g. trial design and how the results/observations were derived [RCTs, non-RCTs, meta-analyses or systematic reviews, clinical case reports, case series]), quantity of data (e.g. number of trials, size of trials, clinical observations only) and consistency of data (e.g. similar or conflicting results across available studies or observations).

The degree of consensus is based on the percentage of Panel vote. For the "uniform consensus" defined in Category 1 and 2A, a majority Panel vote of at least 85% is required. For the "NCCN consensus" defined in Category 2B, a Panel vote of at least 50% (and less than 85%) is required. Lastly, for recommendations where there is string Panel disagreement regardless of the quality of the evidence, NCCN requires a Panel vote of at least 25% to include and designate a recommendation as Category 3. When categories are not specified within the guidelines, the default designation for the recommendation is Category 2A.

Table S4: Attribution of a class of recommendation based on the classification from the American College of Cardiology/American Heart Association Task force [11].

	ASCO	NCCN
Class I	Strong for	Category 1: Based upon high-level evidence, there is uniform NCCN consensus that the intervention is appropriate<br>Category 2A: Based upon lower-level evidence, there is uniform NCCN consensus that the intervention is appropriate.
Class II	Moderate[a]/Weak for	Category 2B: Based upon lower-level evidence, there is NCCN consensus that the intervention is appropriate.<br>Category 3: Based upon any level of evidence, there is major NCCN disagreement that the intervention is appropriate.
Class III	Weak against, Strong against	-

[a]In the ASCO guidelines for pancreatic adenocarcinoma, "moderate" was used as a category of strength of recommendation. ASCO: American Society of Clinical Oncology, NCCN: National Comprehensive Cancer Network guidelines.

References

1. Balshem, H.; Helfand, M.; Schünemann, H.J.; Oxman, A.D.; Kunz, R.; Brozek, J.; Vist, G.E.; Falck-Ytter, Y.; Meerpohl, J.; Norris, S.; et al. GRADE Guidelines: 3. Rating the Quality of Evidence. *J Clin Epidemiol* **2011**, *64*, 401–406.
2. Guyatt, G.H.; Oxman, A.D.; Vist, G.; Kunz, R.; Brozek, J.; Alonso-Coello, P.; Montori, V.; Akl, E.A.; Djulbegovic, B.; Falck-Ytter, Y.; et al. GRADE Guidelines: 4. Rating the Quality of Evidence--Study Limitations (Risk of Bias). *J Clin Epidemiol* **2011**, *64*, 407–415.
3. Guyatt, G.H.; Oxman, A.D.; Kunz, R.; Woodcock, J.; Brozek, J.; Helfand, M.; Alonso-Coello, P.; Glasziou, P.; Jaeschke, R.; Akl, E.A.; et al. GRADE Guidelines: 7. Rating the Quality of Evidence--Inconsistency. *J Clin Epidemiol* **2011**, *64*, 1294–1302.
4. Guyatt, G.H.; Oxman, A.D.; Kunz, R.; Woodcock, J.; Brozek, J.; Helfand, M.; Alonso-Coello, P.; Falck-Ytter, Y.; Jaeschke, R.; Vist, G.; et al. GRADE Guidelines: 8. Rating the Quality of Evidence--Indirectness. *J Clin Epidemiol* **2011**, *64*, 1303–1310.
5. Guyatt, G.H.; Oxman, A.D.; Kunz, R.; Brozek, J.; Alonso-Coello, P.; Rind, D.; Devereaux, P.J.; Montori, V.M.; Freyschuss, B.; Vist, G.; et al. GRADE Guidelines 6. Rating the Quality of Evidence--Imprecision. *J Clin Epidemiol* **2011**, *64*, 1283–1293.
6. Guyatt, G.H.; Oxman, A.D.; Montori, V.; Vist, G.; Kunz, R.; Brozek, J.; Alonso-Coello, P.; Djulbegovic, B.; Atkins, D.; Falck-Ytter, Y.; et al. GRADE Guidelines: 5. Rating the Quality of Evidence--Publication Bias. *J Clin Epidemiol* **2011**, *64*, 1277–1282.
7. Guyatt, G.H.; Oxman, A.D.; Sultan, S.; Glasziou, P.; Akl, E.A.; Alonso-Coello, P.; Atkins, D.; Kunz, R.; Brozek, J.; Montori, V.; et al. GRADE Guidelines: 9. Rating up the Quality of Evidence. *J Clin Epidemiol* **2011**, *64*, 1311–1316.
8. Guyatt, G.; Oxman, A.D.; Sultan, S.; Brozek, J.; Glasziou, P.; Alonso-Coello, P.; Atkins, D.; Kunz, R.; Montori, V.; Jaeschke, R.; et al. GRADE Guidelines: 11. Making an Overall Rating of Confidence in Effect Estimates for a Single Outcome and for All Outcomes. *J Clin Epidemiol* **2013**, *66*, 151–157.
9. Andrews, J.; Guyatt, G.; Oxman, A.D.; Alderson, P.; Dahm, P.; Falck-Ytter, Y.; Nasser, M.; Meerpohl, J.; Post, P.N.; Kunz, R.; et al. GRADE Guidelines: 14. Going from Evidence to Recommendations: The Significance and Presentation of Recommendations. *J Clin Epidemiol* **2013**, *66*, 719–725.
10. Andrews, J.C.; Schünemann, H.J.; Oxman, A.D.; Pottie, K.; Meerpohl, J.J.; Coello, P.A.; Rind, D.; Montori, V.M.; Brito, J.P.; Norris, S.; et al. GRADE Guidelines: 15. Going from Evidence to Recommendation-Determinants of a Recommendation's Direction and Strength. *J Clin Epidemiol* **2013**, *66*, 726–735.
11. Halperin, J.L.; Levine, G.N.; Al-Khatib, S.M.; Birtcher, K.K.; Bozkurt, B.; Brindis, R.G.; Cigarroa, J.E.; Curtis, L.H.; Fleisher, L.A.; Gentile, F.; et al. Further Evolution of the ACC/AHA Clinical Practice Guideline Recommendation Classification System: A Report of the American College of Cardiology/American Heart Association Task Force on Clinical Practice Guidelines. *J Am Coll Cardiol* **2016**, *67*, 1572–1574.
